# A Scoping Review of Magnetic Resonance Modalities Used in Detection of Persistent Postconcussion Symptoms in Pediatric Populations

**DOI:** 10.1177/08830738221120741

**Published:** 2022-11-15

**Authors:** Elena Sheldrake, Brendan Lam, Hiba Al-Hakeem, Anne L. Wheeler, Benjamin I. Goldstein, Benjamin T. Dunkley, Stephanie Ameis, Nick Reed, Shannon E. Scratch

**Affiliations:** 1 Bloorview Research Institute, Toronto, Ontario, Canada; 2 Rehabilitation Sciences Institute, University of Toronto, Toronto, Ontario, Canada; 3 Neuroscience and Mental Health Program, 7979 Hospital for Sick Children, Toronto, Ontario, Canada; 4 Department of Physiology, University of Toronto, Toronto, Ontario, Canada; 5 7978 Centre for Addiction and Mental Health, Toronto, Toronto, Ontario, Canada; 6 Department of Pharmacology and Toxicology, University of Toronto, Toronto, Ontario, Canada; 7 Department of Psychiatry, University of Toronto, Toronto, Ontario, Canada; 8 Institute of Medical Science, University of Toronto, Toronto, Ontario, Canada; 9 Department of Paediatrics, University of Toronto, Toronto, Ontario, Canada

**Keywords:** children, concussion, MRI, neuroimaging, pediatric

## Abstract

Up to 30% of youth with concussion experience PPCSs (PPCS) lasting 4 weeks or longer, and can significantly impact quality of life. Magnetic resonance imaging (MRI) has the potential to increase understanding of causal mechanisms underlying PPCS. However, there are no clear modalities to assist in detecting PPCS. This scoping review aims to synthesize findings on utilization of MRI among children and youth with PPCS, and summarize progress and limitations. Thirty-six studies were included from 4907 identified papers. Many studies used multiple modalities, including (1) structural (n = 27) such as T1-weighted imaging, diffusion weighted imaging, and susceptibility weighted imaging; and (2) functional (n = 23) such as functional MRI and perfusion-weighted imaging. Findings were heterogeneous among modalities and regions of interest, which warrants future reviews that report on the patterns and potential advancements in the field. Consideration of modalities that target PPCS prediction and sensitive modalities that can supplement a biopsychosocial approach to PPCS would benefit future research.

## Concussion and Persistent Postconcussion Symptoms

Mild traumatic brain injury, or concussion, is a serious public health concern. Globally, it is estimated that 6 per 1000 people experience a concussion.^1^ The majority of statistics contributing to global rates originate from North America, particularly in the United States and Canada. In the United States, annual concussion rates among adolescents increased from 19.5% in 2016 to 24.6% in 2021.^2^ In Canada, 110 per 100 000 population reported concussion as their most serious injury, with over half of those injuries related to sports.^3^ Globally, 75% to 90% of traumatic brain injury cases are considered mild,^4^ and 30% of emergency department visits for concussion involve children.^5^ Between 2003 and 2013, emergency department visits for concussion increased 4.4-fold in Ontario each year,^6^ with diversification between sexes. Historically, males experience more concussions compared with females,^3,7^ although females tend to have more severe postconcussion symptoms that take longer to resolve.^7–9^ The presentation of concussion is heterogeneous, but can involve signs and symptoms that are physical (dizziness, headache, fatigue), cognitive (memory problems, attention problems, decreased concentration), behavioral or emotional (irritability, anxiety, depression), as well as sleep problems.^10,11^ The signs and symptoms can impact one's quality of life and ability to participate in necessary and meaningful activities. Currently, a concussion diagnosis is based on history and reported symptoms.

Although most children and youth with concussion experience resolution of their postconcussion symptoms relatively quickly, up to 30% can experience prolonged symptoms lasting for 4 weeks or longer.^12^ Persistent postconcussion symptoms (PPCS) are defined based on time since injury, and include heterogeneous concussion symptom clusters (ie, physical, cognitive, emotional/behavioral, and sleep). In pediatric populations, the range of duration used to define PPCS in published studies varies from 7 days, 10 days, 3 weeks (21 days), 4 weeks (30 days or 1 month), to 3 months.^13–19^ Current clinical guidelines have streamlined the duration criteria to being labeled with prolonged recovery (or having PPCS) to 4 weeks for the pediatric population.^20–22^ Typical assessment for PPCS currently relies on self-reported symptoms of concussion, clinical history, and questionnaire evaluation.^4^ When compared to children with concussion who do not have PPCS, children with PPCS report lower quality of life.^23^ Additionally, PPCS can have resultant impacts on the progression of children and youth into adulthood, including academic, professional, and social domains.^24^ The susceptibility and impact of PPCS on children and youth warrants further investigation into approaches that can assist in detecting PPCS, including structural and functional magnetic resonance modalities.

## Clinical and Experimental Application of Neuroimaging Modalities

For detection of acute and persistent concussion, there is no established universal diagnostic test or marker that can be relied on.^25^ As mentioned, physicians rely primarily on self-reported symptoms from the patient.^26–28^ Although there has been no consensus reached on the use of neuroimaging for diagnostic assessment of concussion or PPCS,^29^ Ontario Neurotrauma Foundation guidelines recommend urgent use of conventional methods (computed tomography [CT] or T1- and/or T2-weighted magnetic resonance imaging [MRI]) in youth with PPCS where a structural brain abnormality is suspected.^22,29^ However, neuroimaging remains an optional step in assessing a potential concussion, as most conventional brain scans show minimal abnormal findings, indicating that current neuroimaging techniques are not yet sensitive enough.^25,30^ In fact, some experts in the field recommend excluding standard neuroimaging (eg, CT) from diagnosis as scans appear typically normal and do not represent an accurate picture of concussion.^30^

In addition to conventional MRI, there are multiple advanced and functional neuroimaging techniques that have shown potential for characterizing subtle microstructural changes that occur from a brain injury.^31^ As MRI continues to evolve and advance over time, certain modalities appear more sensitive to detecting neurologic abnormalities that would otherwise appear normal on a CT scan.^29^ For example, advanced MRI techniques such as functional MRI, perfusion-weighted imaging, and diffusion-weighted imaging show great promise for future clinical diagnosis of concussion, as they identify precise microstructural features of the brain.^32^ Further, as developments in scanner hardware, software, and sensitivities build, conventional MRI could begin to show more detailed and distinct features that may have gone previously undetected. With the variety of definitions and guidelines of PPCS diagnosis and management, neuroimaging biomarkers are an avenue that warrant further research to help address the lack of consensus. To advance the knowledge around PPCS and MRI, it is important to capture the volume and breadth of research in the field of concussion, specifically relating to PPCS and detection.

## Study Objectives

The objective of this scoping review is to summarize and synthesize the literature where MRI is used with pediatric groups experiencing PPCS, in order to better understand if there are any emerging patterns or trends in MRI modalities and progress within this group. This review aims to provide in-depth understanding on MRI modalities that show promise in detecting abnormalities unique to children and youth with PPCS, and therefore a potential utilization of MRI modality as a consistent objective marker for concussion detection.

## Materials and Methods

This scoping review was guided using methodologic frameworks outlined by Arksey and O’Malley^33^ and adapted by Levac, Colquhoun, and O’Brien.^34^ The review is also in accordance with the Joanna Briggs Institute^35^ and the Preferred Reporting Items for Systematic Reviews and Meta-Analysis Extension for Scoping Reviews (PRISMA-ScR).^36^ The scoping review protocol was drafted using the PRISMA-ScR and was registered with the Open Science Framework on July 19, 2021 (https://osf.io/36khq).

### Research Question

This review sought to answer the question, What is the scope of MRI modalities that are used in published research involving children and youth with PPCS?

### Eligibility Criteria

To determine the eligible literature for this review, inclusion and exclusion criteria were set. Studies were included if (1) the population was children and youth (19 years and under) with PPCS (>4 weeks) following a concussion and (2) structural or functional brain MRI modalities were used. The age of 19 years, rather than 18 years, was demarcated using the World Health Organization's definition of adolescence, from ages 10 to 19 years.^37,38^ We defined PPCS according to 3 standards as (1) a concussion diagnosed by a medical professional (ie, physician, emergency department personnel, nurse); (2) diagnosis was determined using standard concussion criteria, such as a Glasgow Coma Scale score ≥13, loss of consciousness ≤30 min, and/or posttraumatic amnesia ≤24 hours, and (3) the presence of at least 1 persistent symptom for a minimum duration of 4 weeks. Four weeks was the chosen minimum duration of persistent symptoms as a majority of American College of Sport Medicine physicians (>50%) defined persistence in youth as greater than 4 weeks,^18^ and the duration is consistent with recent clinical care guidelines.^22^ There was no minimum requirement to the number of persistent symptoms (≥1), as PPCS is currently defined based on time since injury, not symptom count.^30^

Only published works written or translated into the English language were included, because of language restrictions of the reviewers. There was no date limit placed on year of publication to include as many studies as possible; this also allowed for the exploration of any changes in methodology or findings over time. Dissertations, abstracts, conference proceedings and presentations, and non–peer-reviewed publications were not included. A broad range of MRI modalities was considered, including all types of structural and functional brain MRI modalities. Modalities targeting nonneuronal areas, such as magnetic resonance cholangiopancreatography, were not included. Non-MRI modalities were excluded from our study, including but not limited to radiography, CT, positron emission tomography, electroencephalography (EEG), and magnetoencephalography. In studies that included MRI and non-MRI modalities, only MRI-specific details were included.

### Databases and Search Strategy

Four databases were searched: Ovid MEDLINE, CINAHL, PsycINFO, and EMBASE. Search queries were developed using the population, concept, context framework (Supplemental Material Tables 1-4).^39^ Each search was constructed using the Boolean operators AND and OR to optimize search criteria. Additionally, search queries were compiled using the respective database terminology, including MeSH terms and keywords, as well as truncations and adjacencies.

### Study Selection

From the combination of database searches, 4907 papers were identified. EndNote X9^40^ was used to collate all relevant papers and remove any duplicates. After deduplication, 4674 papers met the requirements for screening, and were transferred to Covidence^41^ to facilitate the screening process.

To be included in the review, each paper had to pass 2 screening processes: (1) title and abstract screening and (2) full-text screening. Each paper's title and subsequent abstract was read by 2 independent reviewers (ES, BL), using identical inclusion and exclusion criteria. A third, independent reviewer (HA) assisted in title and abstract screening and acted as arbitrator for any conflicts among papers screened. Prior to full title and abstract screening, a pilot was conducted on the first 20 papers to confirm inclusion criteria was consistent between reviewers. The reviewers agreed to hold a conflict resolution meeting any time the number of papers they disagreed on reached 30 or beyond.

**Figure 1.**
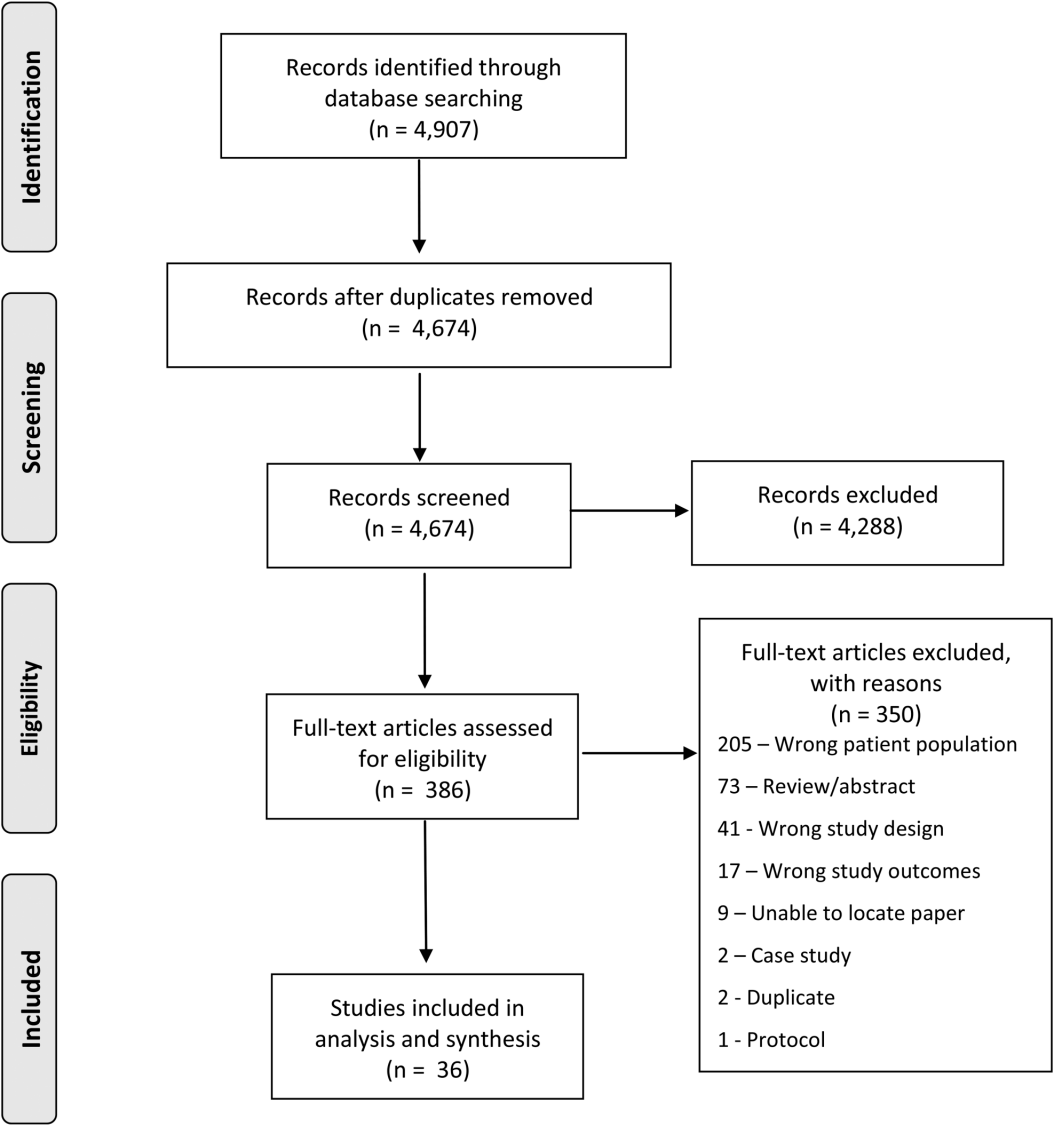
PRISMA diagram.

After title and abstract screening, 386 papers were eligible for full-text screening, which required reviewers to read the paper in its entirety. Thirty-nine papers were included in the review following full-text screening, and 347 papers were excluded (see Figure 1 for PRISMA diagram and reasons for exclusion).

### Charting the Data

Pertinent information for each paper was summarized and collated using Covidence^41^ and Microsoft Excel.^42^ The 3 reviewers met prior to data charting to discuss the design and layout of the spreadsheet. Each reviewer used an identical chart to summarize the data independently. Prior to charting, a pilot run was completed to track the consensus of charting between the 3 reviewers. Each reviewer independently charted the last 6 papers, arranged alphabetically by last name of author, and subsequently met to compare consistency as well as comment on any missing, redundant, or irrelevant areas of the spreadsheet. Following the pilot run and subsequent meeting, the primary reviewer charted all the papers while the other 2 independent reviewers split charting the papers in half. Therefore, each paper was reviewed and charted twice.

The final version of the spreadsheet used for charting was composed of 4 overarching categories: (1) general information, (2) population characteristics, (3) PPCS specifics, and (4) neuroimaging/MRI specifics (see Supplemental Material, Table 5, for detailed charting template). Within the general information category, general study characteristics (eg, study design) were documented. The population characteristics category included study group, control group, and total population characteristics. Within the PPCS specifics category, information collected was concussion history, PPCS criteria and definitions, assessment characteristics, and history of concussion and injury mechanism(s). Finally, the neuroimaging category included subcategories of MRI characteristics (eg, type of modality, region of interest, and measurement parameter), the study variables, and key study findings.

### Analysis

Once all independent charting was complete, each reviewer's charts were amalgamated into a master Microsoft Excel^42^ spreadsheet that listed each reviewer’s answers side-by-side to allow for easy comparison. Using a color-coded system, each reviewer assessed the master chart and made comparative notes between the reviewers’ answers. The coding system was delineated as follows—white: recommend using first reviewer's answers and/or answers are the same between reviewers; green: recommend using second reviewer's answers; orange: minor disagreement between information documented; red: major disagreement between information documented; and blue: recommend merging answers. After color coding, the reviewers met to discuss conflicting answers, and resolve any discrepancies (ie, orange and red data) as well as to consult with each other about analysis of findings. Following discussion, a chart was synthesized to deduce the breadth and span of MRI modalities, summarizing the type of MRI (functional or structural), and further categorized into MRI parameter (cortical thickness/volume, tissue, white matter tracts, cerebral blood flow/volume, resting-state functional MRI, task-based functional MRI, and varieties of parameters). These variables were compared across the study design and types of control group used: PPCS and control group (PPCS and acute concussion group, PPCS vs acute concussion and control group, or no control group), where control group refers to a mild orthopedic injury group or nonclinical control group.

## Results

The results of this scoping review have been organized based on MRI modality, which includes 3 categories: functional MRI or structural MRI. Each MRI modality is further divided by their corresponding clinical distinctions. As the purpose of this scoping review was to gain an understanding of any trends or patterns in MRI findings among youth with PPCS, study results were clustered according to significant and nonsignificant findings within their respective MRI modalities, to consolidate main takeaways (see Table 1 for detailed results). Importantly, the total number of studies contributing to MRI modalities amounts to more than the designated 36 within the review, as some studies included multiple modalities and, as such, may be referenced in more than 1 category.

**Table 1.** Summary of Magnetic Resonance Imaging (MRI) Modality Findings.

Modality	Metric	Author, Year	Region of Interest	Key Findings
Structural	Matter volume/thickness (T1, T2, FLAIR)	Bigler et al, 2013^45^	• Whole brain	• Cortical thickness and gray matter volumes the same between PPCS and control group • No differences in cortical thickness or gray matter volume between TBI severity groups
		Bigler et al, 2018^44^	• Whole brain	• Cortical thickness and gray matter volumes the same between PPCS and control group • Parental PCSI ratings correlated with reduced cortical thickness in PPCS youth
		Iyer et al, 2020^46^	• Structural gray matter	• Cortical thickness and gray matter volumes the same between PPCS and control group • No significant gray matter differences between PPCS and recovered concussion group
		MacDonald et al, 2018^47^	• Whole brain	• Significant decrease in total cortical brain volume in PPCS compared to controls
		MacDonald et al, 2019^48^	• Whole brain	• Significant decrease in total cortical brain volume in PPCS compared to controls
		Ware et al, 2019^49^	• Subcortical brain regions (frontal, cingulum, temporal, parietal, and occipital cortical subregions in each hemisphere)	• Significant increase in left parietal cortical thickness in PPCS group compared to controls
		Yeates et al, 1999^50^	• Whole brain	• Significantly less white matter volume at 7 d and 3 mo postinjury • No significant differences in gray matter volume between groups
		Yeates et al, 2009^51^	• Not specified	• Loss of consciousness in PPCS group positively correlated with MRI intracranial abnormalities
		Zizanovic et al, 2021^52^	• Left dorsolateral prefrontal cortex • Right inferior parietal lobes	• Cortical thickness and gray matter volumes the same between PPCS and control group
	Tissue compound susceptibility (SWI)	Koch et al, 2018^53^	• 28 ROIs comprised of white matter and subcortical gray matter regions	• No significant difference in white matter susceptibility at 6 mo postinjury compared to controls
		Maugans et al, 2011^54^	• Anterior cingulate gyrus • Left dorsolateral prefrontal white matter • Left thalamus	• No significant findings
		Mayer et al, 2019^55^	• Whole brain	• No significant findings
		Ware et al, 2019^49^	• Not specified	• No significant findings
		Ware et al, 2020^56^	• Not specified	• No significant findings
	White Matter Integrity (DWI)	Bartnik-Olson et al, 2014^63^	• Genu, midbody, and splenium of the corpus callosum • Right and left anterior and posterior limbs of the internal capsule	• No significant differences in FA, ADC, and RD over time for any ROI in PPCS relative to controls
		King et al, 2019^62^	• Corticospinal tracts • Uncinate fasciculus • Motor fibers of the corpus callosum	• Mixed results • No significant differences in FA or MD along corpus callosum and left and right corticospinal tract in PPCS relative to controls • Significant decrease in FA and increase in AD in left uncinate fasciculus in PPCS group
		Lancaster et al, 2018^58^	• Genu, splenium, and body of the corpus callosum • Cingulum • Anterior thalamic radiation • Inferior fronto-occipital fasciculus • Superior and inferior longitudinal fasciculus • Anterior and posterior limbs of the internal capsule • Corticospinal tract	• Significant decrease in MD and AD in several white matter tracts 6 mo postconcussion • No significant differences in FA
		MacDonald et al, 2018^47^	• Whole brain	• No DTI results reported
		MacDonald et al, 2019^48^	• 78 regions created from significant clusters in tract-based spatial statistics	• Significant decrease in FA of white matter tracts in left middle frontal gyrus
		Madaan et al, 2021^59^	• Splenium, genu, and body of the corpus callosum • Anterior and posterior limb of the internal capsule • Arcuate fasciculus • Uncinate fasciculus • Left and right frontal white matter tracts	• Weak positive correlation of right uncinate fasciculus FA with verbal comprehension index from WISC-IV
		Manning et al, 2017^60^	• Superior longitudinal fasciculus • Cingulum • Forceps major and minor of the corpus callosum • Uncinate fasciculus • Corticospinal tract • Anterior thalamic radiation • Inferior fronto-occipital and longitudinal fasciculus	• Significant main effect for group differences in corticospinal tract, cingulum, and SLF • Large region of SLF had significant decreases in MD, RD, and AD, and increase in FA 3 mo postinjury
		Maugans et al, 2012^54^	• Anterior cingulate gyrus • Left dorsolateral prefrontal white matter • Left thalamus • Genu, splenium, and body of the corpus callosum • Anterior and posterior limbs of the bilateral internal capsules	• No significant differences in FA, MD, and AD of white matter tracts in PPCS relative to controls
		Satchell et al, 2019^64^	• 48 regions (not specified)	• No significant differences in FA, MD, AD, and RD of white matter tracts in PPCS relative to controls
		Sohn et al, 2020^65^	• Genu, body, and splenium of the corpus callosum	• No significant differences in FA and MD in PPCS relative to controls
		Ware et al, 2019^49^	• Not specified	• No DTI results reported
		Ware et al, 2020^56^	• Not specified	• No significant differences in any white matter tracts investigated
		Yuan et al, 2017^57^	• 90 cortical and subcortical gray matter regions	• Significant increase in global efficiency, and decrease in normalized clustering coefficient, normalized characteristic path length and small-worldness
Functional	BOLD: Resting state	Dona et al, 2017^73^	• 240 regions included in the TT_Daemon human brain atlas	• PPCS individuals had overall lower fractal dimension than controls
		Iyer et al, 2019^67^	• Whole brain	• No significant findings for within-network functional connectivity in 7 brain networks
		Iyer et al, 2019^70^	• Posterior cingulate cortex • Medial prefrontal cortex	• Intraregional connectivity in posterior cingulate cortex was negatively correlated with sleep disturbances in PPCS individuals • Interregional connectivity between posterior cingulate cortex and medial prefrontal cortex were negatively associated with sleep disturbances in PPCS individuals
		Iyer et al, 2020^46^	• Whole brain	• Significant increases in functional connectivity in anterior default mode network and limbic regions
		Lemme et al, 2020^71^	• Whole brain	• Increased functional connectivity in several frontal lobe regions in interior frontal gyrus and cerebellum in PPCS relative to controls
		MacDonald et al, 2018^47^	• Whole brain	• No significant findings in PPCS group
		MacDonald et al, 2019^48^	• Whole brain	• No significant findings
		Mayer et al, 2019^55^	• Whole brain • Left premotor cortex • Bilateral supplemental motor area • Left sensorimotor cortex	• No significant findings
		Manning et al, 2019^60^	• Visual occipital pose • Default mode network • Sensorimotor network • Executive control network • Cerebellar networks	• PPCS groups displayed significant functional activity increases in the occipital pole visual network, sensorimotor network, and cerebellar network, but not in all resting-state networks
		Mutch et al, 2016^69^	• Whole brain	• Significantly greater cerebrovascular response in 0.15% and significantly less response in 5.4% in PPCS individuals
		Mutch et al, 2016^43^	• Whole brain	• No significant differences between PPCS and control groups mean cerebrovascular response • Longitudinally, 4 of 6 PPCS individuals demonstrated abnormal alterations in cerebrovascular response
		Plourde et al, 2020^72^	• Anterior and posterior default mode components	• Significant reduction in connectivity in anterior default mode network of PPCS group
		Stephenson et al, 2020^68^	• Whole brain	• No significant findings in PPCS group
	BOLD: Task-based	Borhorquez-Montoya et al, 2020^74^	• Amygdala	• PPCS individuals had lower amygdala activity during socioemotional processing task relative to healthy controls and downwards trend compared with acute concussion group
		Holmes et al, 2018^75^	• Left and right hippocampus • Anterior and posterior parahippocampus • Lingual gyrus • Lateral occipital cortex	• Increase in number of symptoms of PPCS significantly associated with number of active brain regions • No significant differences identified relative to number or timing of spatial navigation tasks
		Khetani et al, 2019^76^	• Working memory network (including regions in the prefrontal cortex, inferior frontal gyrus, parietal cortex, anterior cingulated, and occipital cortex) • Default mode network (including precuneus and medial prefrontal cortex)	• Decreased activation in posterior cingulate and precuneus during 1-back working memory task in PPCS group • No significance found for working memory task performance between all groups
		Mayer et al, 2019^55^	• Whole brain • Left premotor cortex • Bilateral supplemental motor area • Left sensorimotor cortex	• Hyperactivation in motor circuitry and sensory areas in PPCS participants • Hypoactivation of functional connectivity in PPCS, in addition to decreased areas in prefrontal cortex responsible for cognitive control
	Cerebral blood flow / volume (PWI, pASL)	Barlow et al, 2017^78^	• Whole brain • Regional (bilateral inferior frontal and occipital regions, inferior temporal region, parietal region)	• Higher relative and absolute CBF in PPCS compared to acute concussion • Decrease in CBF was associated with positive recovery trajectory
		Barlow et al, 2021^77^	• Whole brain • Regional (right inferior frontal gyrus, left and right anterior insular gyrus, left superior temporal pole, right superior precentral, and left middle frontal lobe)	• Higher relative and absolute CBF in PPCS compared to acute concussion • Decrease in CBF significantly predicted outcome of recovery status and symptom severity in PPCS youth
		Bartnik-Olson et al, 2014^63^	• Frontal gray matter • Basal ganglia • Thalami • Occipital gray matter • Parietal gray matter	• Significant reductions in CBF and relative CBV in bilateral thalami of PPCS individuals
		Brooks et al, 2018^79^	• Whole brain • Regional (anterior/frontal/temporal regions)	• Hyperfusion in anterior/frontal/temporal regions • Hypofusion in posterior and inferior regions
		Mayer et al, 2019^55^	• Whole brain • Left premotor cortex • Bilateral supplemental motor area • Left sensorimotor cortex	• No significant findings
		Mutch et al, 2016^69^	• Whole brain	• No significant findings
		Stephens et al, 2018^80^	• Whole brain • Left dorsal anterior cingulate cortex • Left insula	• Higher relative cerebral blood flow in left dorsal anterior cingulate cortex and left insula in PPCS compared to controls at both 2 and 6 weeks postconcussion

Abbreviations: AD, axial diffusivity; ADC, apparent diffusion coefficient; BOLD, resting-state blood oxygenation level dependent; CBF, cerebral blood flow; CBV, cerebral blood volume; DTI, diffusion tensor imaging; DWI, diffusion-weighted imaging; FA, fractional anisotropy; FLAIR, fluid attenuated inversion recovery; MD, mean diffusivity; pASL, pseudo-continuous arterial spin labeling; PCSI, Postconcussion Symptom Inventory; PPCS, persistent postconcussion symptoms; PWI, perfusion-weighted imaging; RD, radial diffusivity; ROIs, regions of interest; SLF, superior longitudinal fasciculus; SWI, susceptibility-weighted imaging; TBI, traumatic brain injury.

### General Study Characteristics

This scoping review was composed of 36 studies in total. Studies were published beginning in 1999 to 2021. The studies originated from 4 different countries: United States (n = 19), Canada (n = 15), Australia (n = 1), and India (n = 1). The type of study design varied, although most used a case-control (n = 18) or a controlled cohort structure (n = 13). The remainder of studies used a cohort design without a control group (n = 3) or a randomized controlled trial (n = 2). Studies that used a cohort design compared children or youth with acute concussion to those with persistent or chronic concussion. Controls in the studies were typically developing children or youth (n = 23), youth with mild orthopedic injuries (n = 10), or in the case of one study, siblings of youth with a concussion. Studies either followed a longitudinal (n = 21) or cross-sectional (n = 18) design.

Sample size for PPCS participants varied greatly among studies, ranging from as low as 6 participants^43^ to as high as 219.^44^ Male and female distribution among the PPCS groups was mixed, with the majority of studies having either greater male representation (n = 18) or greater female representation (n = 13). Only 1 study had relatively equal representation of males and females (50% ± 2%). Additionally, 2 studies recruited only male participants, 1 study recruited only female participants, and 1 other study did not document the biological sex of participants. The age range spanned 2-19 years old, with many studies recruiting participants in the late childhood to adolescence stage (8-19 years old). All participants had a concussion diagnosis provided by a health care professional, including physician (n = 22), emergency room personnel (n = 8), athletic trainer (n = 1), or unspecified health care professional (n = 5). Terminology used to describe PPCS varied, but most common terms included persistent or prolonged concussion or postconcussion syndrome. Sports-related concussion was also used frequently in studies that were specific to sport-related concussions.

Twenty-seven studies used structural MRI, and 23 studies included the use of functional MRI. As mentioned, the total number of studies contributing to MRI modalities amounts to more than the designated 36 within the review. Only a handful of studies (n = 8) used only 1 MRI modality. Regarding MRI parameters and identifying factors, many metrics were reported within the reviewed studies, including blood oxygenation following neural activity (n = 13), tissue compound susceptibility (n = 5), white matter integrity (n = 13), cortical thickness or volume (n = 9), and cerebral blood flow / cerebral blood volume (n = 7). Most studies used a 3.0-tesla (T) magnet (n = 32), whereas only a few used a 1.5-T magnet (n = 3). There was 1 study that used both magnetic field strengths (3.0 T and 1.5 T) and 1 study that did not report the field strength of magnet used.

### Structural MRI

Results from structural MRI studies have been divided into MRI modalities to report on the findings related to specific compositions: (1) cortical thickness and volumetric properties, (2) tissue compounds susceptibility, and (3) white matter tract integrity.

### Cortical Thickness and Volumetric Properties (T1, T2)

T1- and T2-weighted imaging are useful MRI modalities that can display a simple, static image of brain composition, showing the contrasts between gray matter, white matter, and fluids. All 36 studies included a T1-weighted sequence for image acquisition, anatomical reference, and/or spatial normalization. Of these studies, 9 included cortical thickness or brain volumetric properties as a part of their objectives and subsequent findings.^44–52^ Regions of interest consisted of whole brain (n = 6), localized regions, including the left dorsolateral prefrontal cortex, right inferior parietal lobes, or subcortical regions in frontal, parietal, temporal, and occipital lobes (n = 2), or unspecified (n = 1; see Table 1 for full region of interest details). Five studies reported significant findings, whereas 4 studies did not find any differences between youth with PPCS and controls.

Five studies reported significant findings related to cortical volume and thickness. Two studies by MacDonald and colleagues^47,48^ investigated volumetric findings longitudinally in youth with PPCS compared with typically developing controls. Both found a significant decrease in total cortical brain volume^47^ and in specific areas of the middle anterior and posterior corpus callosum, and right caudal anterior cingulate cortex^48^ relative to typically developing controls. Further, individuals with PPCS differed significantly from a mild orthopedic injury control group, citing an increase in left parietal cortical thickness.^49^ Additionally, 2 different studies quantifying volumetric brain properties were completed by Yeates and colleagues.^50,51^ The first study discovered that the PPCS group had significantly less white matter volume at both baseline (7 days postinjury) and 3 months postinjury. However, there were no significant differences associated with gray matter between groups.^50^ The second study yielded that LOC reported in children with PPCS was positively associated with MRI intracranial abnormalities (eg, white matter lesions)^51^ compared with orthopedic injury controls.^51^

### Tissue Compounds Susceptibility (Susceptibility-Weighted Imaging, Quantitative Susceptibility Mapping)

Five studies utilized susceptibility modalities, which are useful at detecting venous vasculature and blood products, for enhanced structural composition compared to T1- and T2-weighted options. One study^53^ used quantitative susceptibility mapping, whereas the other 4 used susceptibility-weighted imaging.^49,54–56^ Regions of interest reported were whole brain (n = 1), localized areas, including subcortical gray matter regions, anterior cingulate gyrus, and left dorsolateral prefrontal white matter (n = 2), or unspecified (n = 2). No significant findings were reported across these 5 studies in the prolonged symptoms phase.

### White Matter Tract (Diffusion-Weighted Imaging, Diffusion Tensor Imaging)

Thirteen studies used diffusion-weighted sequences, specifically diffusion tensor imaging, and diffusion kurtosis tensor imaging to investigate white matter connections and microstructural tractography within the brain.^47–49,54,56–60,62–65^ This type of modality is more sensitive to precise white matter details, such as axon structure and integrity. Regions of interest included whole brain (n = 1), localized areas or tracts, including but not limited to the uncinate fasciculus, cingulum, and corticospinal tract (n = 9). Additionally, of the 9 studies that reported on specified regions of interest, 7 of them included the corpus callosum. A few study regions of interest were unspecified (n = 3). Two studies reported the utilization of diffusion-weighted sequences and analysis but did not include any results.^47,49^ Of the 11 remaining studies, 10 measured white matter integrities based on diffusion metrics such as fractional anisotropy, apparent diffusion coefficient or mean diffusivity, axial diffusivity, and radial diffusivity. One study took a tractography-based approach, and applied global and regional network measures including global efficiency (Eglob), mean local efficiency (mean Eglob), modularity (MOD), normalized clustering coefficient (γ), normalized characteristic path length (λ), and small-worldness (σ).^57^ Findings reported from the 11 studies were highly variable; 5 found differences between PPCS and control groups, 5 found no differences, and 1 study had mixed results. Results from studies reporting significant or mixed results are summarized below.

Five studies reported significant differences between PPCS groups and controls in diffusion tensor imaging metrics^48,58–60^ and tractography parameters.^57^ Traditional diffusion tensor imaging metrics demonstrated a significantly decreased mean diffusivity and axial diffusivity in several white matter tracts 6 months postinjury, relative to typically developing controls.^58^ MacDonald and colleagues^48^ also found a significant decrease in fractional anisotropy of the white matter tracts located in the left middle frontal gyrus; however, these differences did not hold after applying Bonferroni-Holm corrections. Manning and colleagues^61^ found significant group differences in the corticospinal tract, cingulum, and superior longitudinal fasciculus (SLF) in youth with PPCS compared with typically developing controls. There were significant decreases in mean diffusivity, radial diffusivity, and axial diffusivity and increases in fractional anisotropy 3 months postinjury along the right superior longitudinal fasciculus^61^ relative to typically developing controls. In another study,^59^ outcomes from the Weschler Intelligence Scale for Children, Fourth Edition (WISC-IV),^61^ were compared to diffusion tensor imaging metrics in children with PPCS relative to typically developing controls. There were positive weak correlations of the right uncinate fasciculus fractional anisotropy with the verbal comprehension index,^59^ even when controlling for age. Using tractography measures, Yuan and colleagues^57^ quantified abnormalities in structural connectivity, implementing a randomized controlled trial of aerobic training versus stretching between groups of PPCS cases and typically developing controls. Following intervention, the PPCS group had a significant increase in Eglob and decrease in γ, λ, and σ compared with typically developing controls. Additionally, improvements in Postconcussion Symptom Inventory symptom scores were correlated with Eglob increase in the aerobic training group and λ decrease in both aerobic training and stretching groups.

One study^62^ described mixed findings between PPCS group diffusion tensor imaging metrics relative to typically developing controls, composed of friends and siblings. Although King and colleagues^62^ noted no differences in fractional anisotropy or mean diffusivity along the corpus callosum and left and right corticospinal tract between the PPCS group and controls, they did note a significant decrease in fractional anisotropy and increase in axial diffusivity in the left uncinate fasciculus in the PPCS group relative to typically developing controls.

### Functional MRI

Functional MRI studies were characterized based on the physiological measurements of brain functioning: (1) blood oxygenation level dependent (BOLD) imaging and (2) cerebral blood flow (eg, perfusion-weighted imaging).

#### Blood oxygenation level dependent imaging

Blood oxygenation level dependent imaging measures functional changes to oxygenation, which infers the activity of brain cells. In studies that utilized blood oxygenation level dependent imaging, the primary types of neuronal activity measurement were divided into resting-state and task-based imaging.^66^ Of the 15 blood oxygenation level dependent imaging studies, 12 studies used resting-state blood oxygenation level dependent whereas 4 used task-based frameworks. One study included used both resting-state and task-based parameters. Most regions of interest consisted of whole brain (n = 8) or network-based analysis, such as the default mode network (n = 4). The remaining studies focused on localized regions (eg, amygdala, prefrontal cortex, sensorimotor cortex, and hippocampus; n = 3).

##### Resting state

Resting-state blood oxygenation level dependent was highly favored in studies using functional modalities. Overall, results from studies varied across regions and networks of interest, measurement parameters, and analyses. Of the 13 resting-state blood oxygenation level dependent studies, 8 studies reported significant differences between groups, and 5 studies did not detect any group differences between PPCS and controls.^47,48,55,67,68^ The 8 studies reporting significant group differences are reported below.

Of the 8 articles, 2 studies^43,69^ had participants undergo carbon dioxide stress testing using resting-state blood oxygenation level dependent to assess cerebrovascular responsiveness in youth with PPCS. In the first study,^70^ patient-specific differences in PPCS individuals were observed in all participants. Voxel-by-voxel comparison of blood oxygenation level dependent cerebrovascular responsiveness for typically developing controls and PPCS patients showed a significantly greater response in 0.15% of the PPCS group, and significantly less responsiveness in 5.4% of the PPCS group. In the second carbon dioxide stress-response study,^43^ 4 of 6 participants with PPCS demonstrated abnormal alterations in cerebrovascular responsiveness, using second-level analysis comparing youth with PPCS to typically developing control cerebrovascular responsiveness longitudinally.^43^

A handful of studies assessed functional connectivity^46,60,67,70–72^ with mixed, but mainly significant, findings. In a study by Iyer and colleagues,^70^ regional homogeneity (intraregional connectivity) and functional connectivity (interregional connectivity) in the posterior cingulate cortex was negatively correlated with sleep disturbances in individuals with PPCS compared to individuals with recovered concussion. Meanwhile, in a randomized control trial by Iyer and colleagues^46^ testing sleep disturbance in individuals with PPCS, functional connectivity was assessed based on levels of melatonin administered. Significant increases in functional connectivity were reported in the anterior default mode network and limbic regions in the PPCS groups administered melatonin, compared with recovered individuals. In addition, Plourde and colleagues^72^ investigated the default mode network specifically and found a significant reduction in connectivity in the anterior default mode network of PPCS groups relative to orthopedic injury controls, particularly in those with multiple concussions.

Lemme and colleagues^71^ compared cohorts of recovered and PPCS groups. It was reported that the PPCS group, compared with recovered controls, exhibited greater functional connectivity in several regions within the frontal lobe including the inferior frontal gyrus and cerebellum, as well as the amygdala and accumbens. Further, in a longitudinal study by Manning and colleagues,^60^ PPCS groups displayed significant functional connectivity increases in the occipital pole visual network, sensorimotor network, and cerebellar network, but not all resting-state networks, relative to typically developing controls.

Two studies^68,73^ focused on abnormalities in neural fluctuations related to resting-state blood oxygenation level dependent. Only 1 study^73^ found significant results in the PPCS group. By analyzing voxelwise fractal dimension, which is hypothesized to show regional changes resulting from brain injury, individuals with PPCS were found to have overall lower fractal dimension than controls.^73^ Specific regions frequently affected included the amygdala, hippocampus, hypothalamus, caudate head, and vermis.^73^

##### Task based

Task-based blood oxygenation level dependent imaging was utilized in 4 studies,^55,74–76^ with varied or conflicting results. The types of tasks varied between studies, including perceptual emotion matching,^74^ spatial navigation tasks,^55,75^ and working memory tasks.^76^ Using perceptual emotion matching, Bohorquez-Montoya and colleagues^75^ aimed to investigate amygdala activation related to social and emotional recognition in youth with PPCS. In addition, psychological measures of general psychological distress, anxiety, depression, and anhedonia were administered, and associations between measures and amygdala activity were assessed. Youth with PPCS exhibited lower amygdala activity on faces vs shape socioemotional processing tasks, relative to typically developing controls and youth with acute concussion. This activity was more apparent in the left amygdala.^74^ However, no differences between PPCS and either group (youth with acute concussion or typically developing controls) were found between amygdala activity and psychological measures.^74^

Two studies^55,75^ sought to evaluate differences between spatial navigation tasks of youth with PPCS, youth with acute concussion, and typically developing controls. Holmes and colleagues^75^ required participants to locate landmarks in a virtual environment. Youth who reported more PPCS also had greater activity in regions of interest compared with typically developing controls, specifically the precuneus, superior frontal gyrus, cerebellum, and frontal orbital cortex.^75^ However, no differences were identified relative to the number or timing of task trials.^75^ Mayer and colleagues^55^ asked participants to respond to multisensory numeric targets using a right-handed button press that corresponded with the target stimulus. In alignment with hypotheses, a failed inhibitory response (hyperactivation) in motor circuitry and sensory areas was exhibited in youth with PPCS relative to controls.^55^ However, contrary to expectations, hypoactivation of functional connectivity in youth with PPCS was observed, in addition to decreased areas in the prefrontal cortex responsible for cognitive control.^55^

Khetani and colleagues^76^ used a working-memory task in children with PPCS compared to children with acute concussion and typically developing controls. A visuospatial n-back working memory task was employed using letter stimuli and measured cortical activity in the dorsolateral prefrontal cortex via blood oxygenation level dependent responses. Decreased activation was found in children with PPCS related to youth with recovered concussion within the posterior cingulate and precuneus during the task, despite similar working task performance between groups.^76^

#### Cerebral blood flow, volume (perfusion-weighted imaging, pseudo-continuous arterial spin labeling)

Seven studies used MRI modalities that identified blood perfusion, including cerebral blood flow and cerebral blood volume. Perfusion-weighted imaging and pseudo-continuous arterial spin labeling measure the relative changes in regional and global blood flow through the brain. Of the 7 studies, 1 study^64^ used dynamic susceptibility contrast-enhanced perfusion-weighted imaging, whereas the rest applied pseudo-continuous arterial spin labeling.^55,69,77–80^ All studies measured relative and/or global/average cerebral blood flow. Most regions of interest reported in studies included both whole brain and specific localized regions (n = 5), although 1 study focused on only localized areas such as the basal ganglia, thalami, premotor cortex, and sensorimotor cortex, and 1 study reported only whole brain findings. Four studies reported differences between youth with PPCS compared to controls, and 3 studies reported no significant differences between groups.

Barlow and colleagues found that individuals with PPCS had higher relative and absolute cerebral blood flow than those with acute concussion^77,78^ and healthy controls.^78^ This was also found in a study by Stephens and colleagues^80^ who found higher relative cerebral blood flow in the left dorsal anterior cingulate cortex and left insula of youth with PPCS compared to typically developing controls at both 2 and 6 weeks postinjury. In addition, 2 other studies found that a decrease in cerebral blood flow significantly predicted the outcome of recovery status and symptom severity in children with PPCS in 77% of cases^77^ and concluded that decrease in cerebral blood flow was associated with positive recovery trajectory.^78^ In contrast, another study reported reduced cerebral blood flow and relative cerebral blood volume in the bilateral thalami of PPCS participants^64^ compared with typically developing controls and is inconsistent with the reports of increased cerebral blood flow as noted. Brooks and colleagues^79^ also noted discordant significant relations of hyperfusion in anterior/frontal/temporal regions and hypofusion in posterior and inferior regions.

Further warranting uncertainty, the remaining studies found no significant differences, or no changes, between PPCS participants’ global cerebral blood flow and individuals with acute concussion^55^ and controls.^55,69,79^

## Discussion

The results of this scoping review provide insight into the present status of neuroimaging in concussion detection among children and youth with PPCS. This review reaffirms the movement toward advanced modalities, particularly evaluating lack of consensus of consistent objective PPCS markers, modalities examining microstructural details and functional neural activity, and the need for a biopsychosocial focus on diagnosis and treatment. Although not the focus of this review, no consistent rationale for regions of interest was established in this literature. There is a lack of consensus within and between modalities, further adding to the variation in findings.

At present, there is no clear consensus on the use of neuroimaging to establish a consistent objective marker of concussion both in the acute and persistent phases. General medical imaging trends over time in both the United States and Canada have seen decreased utilization of CT, and increased utilization of MRI for concussion broadly.^81–83^ With respect to concussion, CT scans are routinely performed upon clinical presentation of head injury, to rule out any macrostructural changes, including brain bleeds, that would be indicative of a moderate or severe traumatic brain injury.^84,85^ After acute phase of concussion, CT scan is less warranted, as it is unable to detect mild or subtle neuroanatomical or neurophysiological change after concussion injury.^86^ Clinically, conventional MRI is used to rule out any other possible neuropathologic reason for prolonged symptom experience, where long-term sequelae are apparent, such as cognitive difficulties and behavioral problems.^84,86^ In most cases, conventional structural MRI is used as a baseline standard for image acquisition, as seen in all studies reviewed. However, in the few studies that sought out differences in cortical thickness or volumetric properties, findings were scattered as the structural MRI was not sensitive enough to detect consistent changes in individuals with PPCS. These variable findings for thickness and volume measures are consistent with PPCS studies from other populations, such as adults and veterans. For example, a study by Rose and colleagues^85^ detected traumatic brain injury abnormalities in only 1.5% of MRIs administered to adult patients in a sports concussion clinic. As a result of these inconsistencies, there has been an increased interest toward using advanced MRI modalities that can detect finer neurologic details. In addition, using combinations of modalities and sequences may be useful to gain comprehensive understanding of neurologic pathology.^87^

Given this shift, a majority of studies used advanced structural imaging techniques (diffusion-weighted imaging or susceptibility-weighted imaging), or functional techniques (functional MRI, perfusion-weighted imaging), which all have increased sensitivity in static and dynamic imaging acquisitions.^84,88^ This is confirmed in the years these studies were undertaken, with newer studies (2014-current) preferring these sensitive, detail-oriented MRI approaches to examine more granular findings in children with PPCS. As MRI research expands, more sensitive structural modalities have begun to identify miniscule structural damage that could be attributable to concussive injury. For example, diffusion tensor imaging has been proven to show brain abnormalities in both acute (one-week)^89^ and persistent (6 months)^90^ concussion in adolescents. In general, diffusion tensor imaging and diffusion kurtosis tensor imaging literature show patterns of decline in axonal and white matter integrity following concussion.^91^ The hope is that patterns will emerge to understand the reorganization of white matter following injury, and the possibility of recovery to axonal damage, particularly if studies increase sample size, or develop biobank standards to look at larger-scale samples.^92^

Traumatic brain injury is an evolving, dynamic process that involves multiple, interrelated neuronal components that can affect both individual neurons and larger networks, and differ among those affected.^93^ Functional imaging techniques show promise in traumatic brain injury detection, with multiple theories postulated for the functional changes in neurologic activity following PPCS. A number of functional MRI studies reported increases in functional connectivity following PPCS, and could be explained by 3 possible theories: (1) “brain reorganization,” in which additional neurons are permanently recruited to an area affected by concussive impact, most likely in the frontal lobe or prefrontal cortex; (2) “neural compensation,” which is thought to recruit additional neurons to an area affected by concussive impact, but is temporary, and therefore compensating short-term; and (3) “latent support hypothesis,” that utilizes extra engagement of cognitive control and attentional resources, rather than permanent or temporary reallocation of neurons.^94^ In addition to functional MRI, research applying other MR modalities to the PPCS population is becoming more commonplace, including magnetization transfer imaging and magnetic resonance spectroscopy that uniquely detects chemical and metabolic composition of scanned tissues that may indicate abnormal brain activity. In a review conducted by Eisele and colleagues,^95^ magnetic resonance spectroscopy was identified as detecting metabolic changes in the brain in adults with acute or prolonged concussion (≤90 days postconcussion), even with a negative CT and/or structural MRI result. Particularly, brain metabolites *N*-acetyl-aspartate, glutamate, and choline showed considerable changes between healthy controls and concussed individuals.^95^ Although brain metabolite creatine was not a suitable marker, 3 metabolites signaled significant changes that conventional MRI failed to distinguish.^95^ Aside from MRI, non-MR functional instruments, such as EEG and magnetoencephalography both show evidence of neurophysiological changes in individuals with both acute and chronic concussions and are sensitive to rapid neurologic alterations.^96,97^

One issue consistent across MRI modalities is that findings often show significance at a group level, but rarely at an individual level.^29,91,98^ Specific to concussion, the variability in symptoms creates a difficult barrier to pinpointing specific biomarkers when individuals present with such differing sequalae and is reflective of the contradictory results across modalities.^91^ Given the heterogeneity of postconcussion symptoms across individuals, it is possible that distinct subgroups of concussion and PPCS will emerge over time, to help define concussion prognosis and sequelae.^99^ Perhaps with advancements in concussion detection research, specific modalities will become favored depending on PPCS subgroups. To add, combinations of modalities and sequences rather than 1 MRI modality would assist in gaining a more comprehensive understanding of both anatomical and functional characteristics of an individual with a concussion.^100^ Although this may be time consuming and costly, it is beneficial to gather as much information about neurobiological composition and function that would otherwise be missed by completing only 1 kind of MRI modality.^92^

As this review focused solely on pediatric populations, it is important to discuss the factors associated with persistent concussion and neuroimaging issues specific to infants, children, and youth. First, there is a significant number of youth concussions that go unreported, especially in young athletes dealing with sport-related concussions.^101–103^ Second, children and youth are at a critical period of brain maturation, in which neurologic injury can have much larger, lasting impacts than those experiencing a concussion in adulthood.^104^ Therefore, even though older children are more likely to experience persistent symptoms, infants and young children are still vulnerable to severe impacts of concussion.^105^ Third, as children and youth are still developing, it is possible that the identification of concussive injury is different depending on their age group and developmental stage.^98^ For example, myelination begins in late pregnancy, but is not fully complete until late adolescence or early adulthood.^98^ Therefore, depending on age, a lack of microstructural axonal integrity could not always be reflective of concussion as it could be related to a child's development stage. Fourth, infant concussion is widely understudied and underrepresented in current literature, even though infants represent a high-risk group for sustaining concussion.^106,107^ In particular, there is a paucity in the exploration of PPCS within infant populations. In fact, despite the broad inclusion criteria for this review, no studies were identified that focused on infants (<2 years old). Hence, it is difficult to understand the level of severity of concussion injury, as well as the presentation and duration of symptoms within infants.

Although this review focused on pediatric populations, no important distinctions were acknowledged between sex and gender in any studies, and only a minority of studies represented equal male and female distribution. There are important disparities between sexes and genders in children and youth that should be considered in future studies, as it may impact both neuroimaging results and study participation among males and females. Neuroimaging studies report differences in brain abnormalities between males and females, such as more severe white matter damage than their male counterparts, albeit rare.^87,108^ In addition, it has been hypothesized that females are biologically predisposed to lower biomechanical thresholds, causing greater susceptibility to concussions.^8^ It is also well reported that males have a greater rate of hospitalization due to concussion, although females are at higher risk of sustaining a concussion and subsequent persistent symptoms.^109^ This may be due to the greater “risk-taking” behaviors that males often exhibit, and increased symptom reporting by females as it is more socially acceptable to exhibit vulnerability in females compared with males.^8^ Additionally, studies included were very diverse in sample size and control groups (if any). Broad differences in sample size and control groups add to the conversation of factors that could create discrepancies among studies and impact study findings and subsequent conclusions. In an ideal world, prospective studies that include preinjury MRI as a baseline would provide the best comparison measure for the true impact of concussion, albeit difficult to execute as one cannot predict who will get a concussion. However, greater conformity among control groups would assist in current obfuscation, and lack thereof is acknowledged as a limitation in the field.

### Strengths and Limitations

This scoping review provided the first synthesis of the existing literature on pediatric PPCS using structural and functional magnetic resonance modalities. As many scoping reviews only have 2 reviewers, the additional third reviewer is a strength of our approach. Three reviewers all completed title and abstract screening, full-text screening, charting, and analysis (ES, BL, HA). Having 3 reviewers allowed for a thorough, detailed, and unified review process, as meetings to discuss discrepancies, consensus, and missing details were completed weekly. A greater number of reviewers is preferred when conducting scoping reviews, so long as calibration exercises are regularly completed, as was the case with this review.^110^ In addition, a comprehensive database search was conducted that encompassed a range of disciplines that could house neuroimaging and PPCS literature. In doing so, a broad overview of existing research in the field was completed, commenting on the wide range of modalities, findings, and study designs.^36^

As well, terminology was inclusive of the variety of definitions and criteria that could include or characterize PPCS, including synonyms of chronic, long-term, persistent, and prolonged. Although terminology of PPCS was widely inclusive, the sheer amount of terminology, varying definitions, and differing criteria for both concussion and PPCS is a limitation in itself. There is no standard, consensual definition for PPCS, which causes uncertainty in both research and practice.^18^ First, diagnostic criteria hinges on age and duration of symptoms; children and adolescents (≤18 years old) must experience symptoms for 4 weeks or longer,^20–22^ compared with 3 months in adults.^18^ The type and number of symptoms can vary greatly from person to person, making it hard to diagnose based solely off symptom presentation.^18^ Second, terminology has shifted across categorization systems, further leading to inconsistencies; for example, the fourth edition of the *Diagnostic and Statistical Manual of Mental Disorders* (*DSM-IV*) included the term “post-concussional disorder,”^111^ whereas the more recent fifth edition (*DSM-V*) has dropped the name entirely.^112^ The lack of definitive, conclusive terminology and diagnostic criteria certainly warrants concern when investigating this ambiguous group, and is reasonable to suspect that some studies in this review could have been overlooked, or included erroneously as a result. As literature in the field of concussion continues to evolve, so do the opinions and expertise. At this moment, a broad range of terminology to capture the heterogeneity of the field was the optimal approach; however, a long-term goal in the field should be to gain consensus to make research and diagnostic processes more streamlined.

Including studies that captured a range of postconcussion symptoms and considered participants to have PPCS with a minimum of 1 persisting postconcussion symptom is both a strength and limitation to the study. In doing so, the number of studies captured was maximized, but it created a very broad definition of PPCS itself, therein also reducing the sensitivity of studies included. As this was a scoping review, the purpose was to synthesize and summarize the existing literature on the topic, not to evaluate the quality of evidence, designs, and methods chosen, and therefore is lacking in any critical appraisal or formal quantitative synthesis.^39^ In addition, there is a risk of bias, especially selection bias, as with any scoping review, and although hand-searching of references was completed, gray literature was omitted from the scoping review. This creates a limitation in restricting the possible unpublished or non–peer-reviewed studies that could add to the review findings and overall scope of the literature. Finally, the adaptations to Arksey and O’Malley's^33^ seminal scoping review guidelines provided by Levac and colleagues^34^ included an additional step to the process: consultation. This stage, although optional, proposes that consultation should be an essential inclusion to the methodology, and calls for stakeholder engagement on preliminary findings and how to implement and disseminate to end users.^34^ This step was omitted from this review and should be included in future reviews to enable optimal knowledge translation of these findings within the field.

### Future Directions

The progression of acute concussion to PPCS is poorly understood, including both factors that cause this shift to persistent symptoms in only some individuals, as well as how to objectively detect and predict who will inherit these long-lasting effects beyond the use of self-reported symptoms.^113,114^ Prediction of PPCS using MRI is an important future focus as neuroimaging modalities may be able to target specific biomarkers that predict the possible development of PPCS.^77,115^ With more longitudinal, prospective studies designed to track changes in neuroanatomical and neurofunctional status, it is hoped that key regions of interest, metabolites, or networks, to name a few, that predict development of chronic symptoms can be identified.^29,98,116,117^

A biopsychosocial approach to diagnosis and treatment of PPCS in children and youth is a favorable option to incorporate a multifaceted plan that complements the heterogeneity of symptom presentation. Ventresca^118^ proposed that qualitative research may be useful to assist in gaining a fuller, more comprehensive understanding of individual concussion, that could grasp at the deeper, psychosocial underpinnings of concussion not captured quantitatively. From qualitative inquiry, further information on the social and emotional side of persistent concussion could be captured that could lead to greater clarification of future neuroimaging studies of networks or regions of interest.^118,119^ Biopsychosocial models align with a multidisciplinary approach to diagnosis, treatment, and management, which could also be a propitious idea to tackle the individuality and heterogeneity of PPCS symptoms. Multidisciplinary approaches could incorporate a combination of neuroimaging in conjunction with neuropsychological measures, and self-report questionnaires, that includes both objective biomarkers and standardized scores, in addition to more subjective profiles.^115,120,121^ Doing so would paint a clearer picture as to a particular presentation, in addition to consistent biological targets.

This scoping review's purpose was to synthesize and summarize the MRI modalities used in existing research involving children and youth with PPCS and their subsequent findings. Consequently, a future systematic review would be an appropriate direction to critically appraise, analyze, and amalgamate the quality of the studies in methodology, design, and results.^122^ Systematic reviews are unbiased in reasoning, and therefore are more reliably used to influence health care decisions^123^ and, as such, this scoping review could act as a precursor to delve further into the effectiveness of the studies.

## Conclusions

This study adds to the advancing field of concussion, by consolidating the heterogeneous literature of PPCS and MRI, which will help disentangle and pinpoint promising modalities and areas of focus moving forward. Children and youth are at risk for concussion and subsequent PPCS. Neuroimaging could be a pivotal component to diagnosis concussion and PPCS in the future. Continued research to detect brain biomarkers exclusive to concussion and/or PPCS is needed. Although MRI does not yet have any solidified brain biomarkers, both advanced structural and functional modalities, such as diffusion tensor imaging and functional MRI, show glimpses of potential at pinpointing patterns in complex microstructural and network damage resulting from concussion. As research in this area continues to accelerate, there is great hope that further discoveries will emerge to break down the complexities in symptoms and diagnosis, to create practical and invaluable treatment and management for vulnerable children and youth impacted by concussion and related poor health outcomes.

## Supplemental Material

sj-docx-1-jcn-10.1177_08830738221120741 - Supplemental material for A Scoping Review of Magnetic Resonance Modalities Used in Detection of Persistent Postconcussion Symptoms in Pediatric Populations Click here for additional data file. Supplemental material, sj-docx-1-jcn-10.1177_08830738221120741 for A Scoping Review of Magnetic Resonance Modalities Used in Detection of Persistent Postconcussion Symptoms in Pediatric Populations by Elena Sheldrake, Brendan Lam, Hiba Al-Hakeem, Anne L. Wheeler, Benjamin I. Goldstein, Benjamin T. Dunkley, Stephanie Ameis, Nick Reed and Shannon E. Scratch in Journal of Child Neurology
