# Supplementary material for: A Scoping Review of Magnetic Resonance Modalities Used in Detection of Persistent Postconcussion Symptoms in Pediatric Populations
Source: J Child Neurol. 2022 Nov 15;38(1-2):85–102. doi: 10.1177/08830738221120741 (PMC10061627; doi:10.1177/08830738221120741)
Supplement: sj-docx-1-jcn-10.1177_08830738221120741 - Supplemental material for A Scoping Review of Magnetic Resonance Modalities Used in Detection of Persistent Postconcussion Symptoms in Pediatric Populations [file sj-docx-1-jcn-10.1177_08830738221120741.docx]

**Supplemental Material**

**Table 1: Ovid MEDLINE Search Outline**

| # | Search Query | Results |
| --- | --- | --- |
| 1 | Post-Concussion Syndrome/ | 1175 |
| 2 | brain injuries, traumatic/ or brain concussion/ or brain injury, chronic/ | 16603 |
| 3 | (post-concuss* or post concuss* or postconcuss).tw,kf | 3196 |
| 4 | ((persist* or prolonged or chronic or long-term) adj3 (brain injur* or concuss* or head injur*)).tw,kf. | 6264 |
| 5 | (mtbi or mild trauma* adj3 brain injur* or mild TBI).tw,kf | 41758 |
| 6 | exp adolescent/ or exp child/ or exp infant/ or (infant disease* or childhood disease*).ti,ab,kf. Or (adolescen* or babies or baby or boy? Or boyfriend or boyhood or girlfriend or girlhood or child* or girl? Or infan* or juvenil* or kid? Or minors or minors* or neonat* or neo-nat* or newborn* or new-born* or paediatric* or peadiatric* or pediatric* or perinat* or preschool* or puber* or pubescen* or school* or teen* or toddler? Or underage? Or under-age? Or youth*).ti,ab,kf. Or (pediatric* or paediatric* or infan* or child* or adolescen* or young).jn,jw. Or (pediatric* or paediatric* or infan* or child* or adolescen* or young).in. | 5097521 |
| 7 | diagnostic imaging/ or neuroimaging/ or diffusion tensor imaging/ or functional neuroimaging/ or brain mapping/ | 155373 |
| 8 | tomography/ or magnetic resonance imaging/ or diffusion magnetic resonance imaging/ or diffusion tensor imaging/ or echo-planar imaging/ or fluorine-19 magnetic resonance imaging/ or magnetic resonance angiography/ or magnetic resonance imaging, cine/ or multiparametric magnetic resonance imaging/ | 475895 |
| 9 | (neuroimage* or magnetic resonance imag* or fMRI or MRI or brain scan* or brain map* or tomograph* or echo-planar imag* or multiparametric magnetic resonance imag* or blood oxygen level dependent* or BOLD).tw,kf. | 844326 |
| 10 | 1 or 2 or 3 or 4 or 5 | 49455 |
| 11 | 7 or 8 or 9 | 1103098 |
| 12 | 6 and 10 and 11 | 2567 |
| 13 | Post-Concussion Syndrome/dg [Diagnostic Imaging] | 39 |
| 14 | Brain Concussion/dg [Diagnostic Imaging] | 568 |
| 15 | 13 or 14 | 589 |
| 16 | 6 and 15 | 299 |
| 17 | 12 or 16 | 2646 |

**Table 2: PsychInfo Search Outline**

| # | Search Query | Results |
| --- | --- | --- |
| 1 | traumatic brain injury/ or brain injuries/ or brain concussion/ | 20763 |
| 2 | (post-concuss* or post concuss* or postconcuss).tw | 1080 |
| 3 | (mtbi or mild trauma* brain injur* or mild TBI).tw | 3781 |
| 4 | ((persist* or prolonged or chronic or long-term) adj3 (brain injur* or concuss* or head injur*)).tw | 971 |
| 5 | post-concussion syndrome.tw | 222 |
| 6 | 1 or 2 or 3 or 4 or 5 | 21490 |
| 7 | adolescent development/ or childhood development/ or pediatrics/ or exp Congenital Disorders/ or child characteristics/ or child abuse/ or exp child welfare/ or chronically ill children/ or child neglect/ or child psychiatry/ or child psychopathology/ or exp child care/ or (pediatric* or paediatric* or child* or newborn* or congenital* or infan* or baby or babies or neonat* or pre term or preterm* or premature birth or NICU or preschool* or pre school* or kindergarten* or elementary school* or nursery school* or schoolchild* or toddler* or boy or boys or girl* or middle school* or pubescen* or juvenile* or teen* or youth* or high school* or adolesc* or prepubesc* or pre pubesc*).mp. or (child* or adolesc* or pediat* or paediat*).jn. | 1326238 |
| 8 | neuroimaging/ or tomography/ or diffusion tensor imaging/ | 23162 |
| 9 | magnetic resonance imaging/ or functional magnetic resonance imaging/ | 44272 |
| 10 | (neuroimag* or brain imag* or brain scan* or brain map* or functional magnetic resonance imag* or fMRI or MRI or magnetic resonance imag* or arterial spin label* or magnetic resonance angiograph*).tw. | 107127 |
| 11 | 8 or 9 or 10 | 114252 |
| 12 | 6 and 7 and 11 | 688 |

**Table 3: CINAHL Search Outline**

| # | Search Query | Results |
| --- | --- | --- |
| 1 | (MH “Postconcussion Syndrome”) | 828 |
| 2 | (MH “Brain Concussion+”) | 5492 |
| 3 | TI (mild TBI or mTBI or mild trauma* brain injur*) OR AB (mild TBI or mTBI or mild trauma* brain injur*) | 2610 |
| 4 | TI (post-concuss* or postconcuss* or post concuss*) OR AB (post-concuss* or postconcuss* or post concuss*) | 1449 |
| 5 | TI (chronic brain injur*) OR AB (chronic brain injur*) | 39 |
| 6 | TI ((persist* or prolonged or chronic or long-term) N3 (brain injur* or concuss* or head injur*) OR AB ((persist* or prolonged or chronic or long-term) N3 (brain injur* or concuss* or head injur*)) | 1165 |
| 7 | S1 OR S2 OR S3 OR S4 OR S5 OR S6 | 8139 |
| 8 | (pediatric* or paediatric* or child* or newborn* or congenital* or infan* or baby or babies or neonat* or “pre-term” or preterm or “premature birth*” or NICU or preschool* or “pre-school*” or kindergarten* or “elementary school*” or “nursery school*” or schoolchild* or toddler* or boy or boys or girl* or “middle school*” or pubescen* or juvenile* or teen* or youth* or “high school*” or adolesc*or prepubesc* or “pre-pubesc*” or (MH "Child+") OR (MH "Adolescence+") OR (MH "Minors (Legal)") or "(MH "Child Abuse, Sexual") OR (MH "Child Behavior Disorders+") OR (MH "Child, Medically Fragile") OR (MH "Child Day Care") OR (MH "Child Behavior+") OR (MH "Child Mortality") OR (MH "Child Passenger Safety") OR (MH "Child Development Disorders, Pervasive+") OR (MH "Child Custody") OR (MH "Child Abuse+") OR (MH "Child Nutritional Physiology+") OR (MH "Child Behavior Checklist") ) OR SO ( child* or pediatric* or paediatric* or adolescent ) | 1389751 |
| 9 | (MH "Tomography, X-Ray") OR (MH "Neuroradiography") OR (MH "Cerebral Angiography") | 10046 |
| 10 | (MH "Magnetic Resonance Imaging") OR (MH "Magnetic Resonance Angiography") | 130405 |
| 11 | TI (neuroimag* or neural imag* or brain imag* or brain scan* or ((functional) N3 (magnetic resonance imag* or MRI)) or fMRI or magnetic resonance angiograph* or arterial spin label*)) OR ( AB(neuroimag* or neural imag* or brain imag* or brain scan* or ((functional) N3 (magnetic resonance imag* or MRI)) or fMRI or magnetic resonance angiograph* or arterial spin label*) ) | 25761 |
| 12 | S9 OR S10 OR S11 | 146507 |
| 13 | S7 AND S8 AND S12 | 209 |

**Table 4: EMBASE Search Outline**

| # | Search Query | Results |
| --- | --- | --- |
| 1 | exp post-concussion syndrome/ | 61676 |
| 2 | brain injuries, traumatic/ or brain concussion/ or brain injury, chronic/ | 4809 |
| 3 | (post-concuss* or post concuss* or postconcuss).tw,kw | 9813 |
| 4 | ((persist* or prolonged or chronic or long-term) adj3 (brain injur* or concuss* or head injur*)).tw,kw | 3052 |
| 5 | (mtbi or mild trauma* brain injur* or mild TBI).tw,kw | 2526 |
| 6 | 1 or 2 or 3 or 4 or 5 | 66235 |
| 7 | exp adolescence/ or exp adolescent/ or exp child/ or exp childhood disease/ or exp infant disease/ or (adolescen* or babies or baby or boy? or boyfriend or boyhood or girlfriend or girlhood or child* or girl? or infan* or juvenil* or juvenile* or kid? or minors or minors* or neonat* or neo-nat* or neo-nat* or newborn* or new-born* or paediatric* or peadiatric* or pediatric* or perinat* or preschool* or puber* or pubescen* or school or school child* or school* or schoolchild* or schoolchild* or teen* or toddler? or underage? or under-age? or youth*).ti,ab,kw. | 6132309 |
| 8 | exp neuroimaging/ | 145804 |
| 9 | nuclear magnetic resonance imaging/ or arterial spin labeling/ or cine magnetic resonance imaging/ or diffusion tensor imaging/ or diffusion weighted imaging/ or dynamic contrast-enhanced magnetic resonance imaging/ or echo planar imaging/ or fluorine magnetic resonance imaging/ or functional magnetic resonance imaging/ or interventional magnetic resonance imaging/ or magnetic resonance angiography/ or magnetic resonance elastography/ or multiparametric magnetic resonance imaging/ or perfusion weighted imaging/ or susceptibility weighted imaging/ or whole body mri/ | 973052 |
| 10 | (magnetic resonance imag* or MRI or fMRI or functional MRI or blood oxygen level dependent or BOLD or neuroimag* or brain map* or neural imag* or brain imag* or diffusion MRI or diffusion weighted imag* or diffusion tensor imag* or echo planar imag* or susceptibility weighted imag* or arterial spin label*).ti,ab,kw. | 749750 |
| 11 | 8 or 9 or 10 | 1133417 |
| 12 | 6 and 7 and 11 | 2503 |

**Table 5: Study Charting Template**

| **General Information** | | | | | | **Study Design** | |
| --- | --- | --- | --- | --- | --- | --- | --- |
| Count | Author | Year | Title | Country | Objectives | Design | Longitudinal or Cross-Sectional? |

| **Population** | | | | | | | | | |
| --- | --- | --- | --- | --- | --- | --- | --- | --- | --- |
| **Study Group** | | | | **Control Group** | | | | **Total** | |
| Group | Biological Sex | Age (µ, sd) | n | Group | Biological Sex | Age (µ, sd) | n | Age Range | n |

| **PPCS** | | | | | | | |
| --- | --- | --- | --- | --- | --- | --- | --- |
| **PPCS Diagnostic Criteria/Definitions** | | | | | | **Assessment Characteristics** | |
| # Prior Concussion | Mechanism of Injury | Concussion Terminology | Diagnosis | Diagnostics | Duration | Assessment | Assessment Time After Injury |

| **Neuroimaging** | | | | | | | | | |
| --- | --- | --- | --- | --- | --- | --- | --- | --- | --- |
| **MRI Characteristics** | | | | | **Variables** | | | **Key Findings** | |
| MRI Type | ROI | Measurement Outcome | Magnet | Scan Time After Injury | Independent Variable | Dependent Variable | Analysis | Association | Other |
